# RETRACTION: **Calycosin Inhibits Hepatocyte Apoptosis in Acute Liver Failure by Suppressing the TLR4/NF‐κB Pathway: An In Vitro Study**

**DOI:** 10.1002/iid3.70234

**Published:** 2025-07-30

## Abstract

**RETRACTION:** L. Chang, A. Zhang, W. Liu, P. Cao, L. Dong, and X. Gao, “Calycosin Inhibits Hepatocyte Apoptosis in Acute Liver Failure by Suppressing the TLR4/NF‐κB Pathway: An In Vitro Study,” *Immunity, Inflammation and Disease* 11, no. 7 (2023): e935, https://doi.org/10.1002/iid3.935.

The above article, published online on July 12, 2023, in Wiley Online Library (wileyonlinelibrary.com), has been retracted by agreement between the journal Editor‐in‐Chief, Marc Veldhoen; and John Wiley & Sons, Ltd. The retraction was agreed upon following an investigation into concerns raised by a third party regarding unexpected similarity between the flow cytometry data in Figure 2C of this article and data published earlier in a separate article by a different group of authors. Inconsistencies were also observed in the western blot presented in Figure 5A of this article. The authors provided supporting data for the flow cytometry plots in Figures 2C and 7C; however, the data did not align with the figures presented in the published article. Additionally, they did not address or provide any data in response to concerns regarding the western blot. Given these issues and the lack of adequate supporting data, the editors find the article's results and conclusions to be unreliable. The authors were informed of the decision to retract but did not respond to requests for comment.

**RETRACTION:** L. Chang, A. Zhang, W. Liu, P. Cao, L. Dong, and X. Gao, “Calycosin Inhibits Hepatocyte Apoptosis in Acute Liver Failure by Suppressing the TLR4/NF‐κB Pathway: An In Vitro Study,” *Immunity, Inflammation and Disease* 11, no. 7 (2023): e935, https://doi.org/10.1002/iid3.935.

The above article, published online on July 12, 2023, in Wiley Online Library (wileyonlinelibrary.com), has been retracted by agreement between the journal Editor‐in‐Chief, Marc Veldhoen; and John Wiley & Sons, Ltd. The retraction was agreed upon following an investigation into concerns raised by a third party regarding unexpected similarity between the flow cytometry data in Figure 2C of this article and data published earlier in a separate article by a different group of authors. Inconsistencies were also observed in the western blot presented in Figure 5A of this article. The authors provided supporting data for the flow cytometry plots in Figures 2C and 7C; however, the data did not align with the figures presented in the published article. Additionally, they did not address or provide any data in response to concerns regarding the western blot. Given these issues and the lack of adequate supporting data, the editors find the article's results and conclusions to be unreliable. The authors were informed of the decision to retract but did not respond to requests for comment.

